# Clinical Significance of M1/M2 Macrophages and Related Cytokines in Patients with Spinal Tuberculosis

**DOI:** 10.1155/2020/2509454

**Published:** 2020-05-20

**Authors:** Liang Wang, Xiaoqian Shang, Xinwei Qi, Derong Ba, Jie Lv, Xuan Zhou, Hao Wang, Nazierhan Shaxika, Jing Wang, Xiumin Ma

**Affiliations:** ^1^State Key Laboratory of Pathogenesis, Prevention and Treatment of High Incidence Diseases in Central Asian, Clinical Medical Research Institute of First Affiliated Hospital of Xinjiang Medical University, Urumqi, Xinjiang, China 830011; ^2^Department of Spinal Surgery, People's Hospital of Xinjiang Uygur Autonomous Region, Urumqi, Xinjiang, China 830001; ^3^Department of Respiratory Medicine, First Affiliated Hospital of Xinjiang Medical University, Urumqi, Xinjiang, China 830011; ^4^Clinical Laboratory Center, Tumor Hospital Affiliated to Xinjiang Medical University, Urumqi, Xinjiang, China 830011

## Abstract

**Background:**

Macrophages are important immune cells involved in *Mycobacterium tuberculosis* (*M.tb*) infection. To further investigate the degree of disease development in patients with spinal tuberculosis (TB), we conducted research on macrophage polarization.

**Methods:**

Thirty-six patients with spinal TB and twenty-five healthy controls were enrolled in this study. The specific morphology of tuberculous granuloma in spinal tissue was observed by hematoxylin-eosin (H&E) staining. The presence and distribution of bacilli were observed by Ziehl-Neelsen (ZN) staining. Macrophage-specific molecule CD68 was detected by immunohistochemistry (IHC). M1 macrophages play a proinflammatory role, including the specific molecule nitric oxide synthase (iNOS) and the related cytokine tumor necrosis factor-*α* (TNF-*α*) and interferon-*γ* (IFN-*γ*). M2 macrophages exert anti-inflammatory effects, including the specific molecule CD163 and related cytokine interleukin-10 (IL-10). The above markers were all detected by quantitative real-time PCR (RT-PCR), enzyme-linked immunosorbent assay (ELISA), and IHC.

**Results:**

Typical tuberculous granuloma was observed in the HE staining of patients with spinal TB. ZN staining showed positive expression of Ag85B around the caseous necrosis tissue and Langerhans multinucleated giant cells. At the same time, IHC results indicated that CD68, iNOS, CD163, IL-10, TNF-*α*, and IFN-*γ* were expressed around the tuberculous granuloma, and their levels were obviously higher in close tissue than in the distant tissue. RT-PCR and ELISA results indicated that IL-10, TNF-*α*, and IFN-*γ* levels of TB patients were also higher than those of the healthy controls.

**Conclusion:**

The report here highlights that two types of macrophage polarization (M1 and M2) are present in the tissues and peripheral blood of patients with spinal TB. Macrophages also play proinflammatory and anti-inflammatory roles. Macrophage polarization is involved in spinal TB infection.

## 1. Introduction

*Mycobacterium tuberculosis* (*M.tb*) is a type of intracellular parasitic bacteria that causes tuberculosis (TB). TB is one of the most threatening infectious diseases to human beings, and patients with TB account for about one-third of the world's population. With the continuous spread of drug-resistant *M.tb*, the incidence of diseases caused by Human Immunodeficiency Virus (HIV)/*M.tb* coinfection is increasing [1, 2]. TB is mainly transmitted through the respiratory tract. Once infected, it can spread to multiple systems through the blood, such as the skeletal system and the digestive system. Extrapulmonary TB accounts for about 50% of TB patients. Among them, bone and joint TB are most common in the spine. The *M.tb* of the primary lesion of spinal TB can directly spread to the edge of the vertebral body through the blood, lymphatic vessels, and pleural and lymph node lesions [3, 4], which further cause the destruction of vertebral body or intervertebral discs, spinal deformity and dysfunction, and even paraplegia and death [5, 6]. Hypersensitivity and immune responses are involved in *M.tb* infection, resulting in three basic pathological changes, including exudation, hyperplasia, and degeneration/necrosis. In the early stage of the disease or when the body has low resistance, accompanied by a large amount of bacteria, strong virulence, and strong hypersensitivity reaction, the pathological manifestations are mainly serous or serous cellulitis, which are featured by a large number of neutrophil infiltration and macrophage migration to the lesion under the action of inflammatory factors, thus clearing *M.tb* [7]. If early control is not appropriate, tuberculous granuloma, the characteristic structure of TB infection, will appear [8]. Tuberculous granuloma is formed by macrophages, epithelioid cells, Langerhans multinucleated giant cells, lymphocytes, and a few fibroblasts, and its main pathological manifestation is tissue hyperplasia. Moreover, as the disease progresses further, caseous necrosis can eventually occur.

When the tuberculosis infection is in the early stages, the disease progresses slowly and the bone destruction is mild. At this time, conventional nonsurgical treatment can be used to relieve it. However, when the progress of the disease is obvious, the vertebral body and the vertebrae are broken, the vertebral body is damaged obviously, the bone defect is serious, and the stability of the vertebral body is damaged. This indicates that the course of disease is in the stage of disease development. At this time, the patient needs surgical intervention to prevent the disease from further worsening.

Macrophages are the main effector cells to kill and clean *M.tb*, but they are also the host cells of *M.tb* infection. They participate in the whole process of the occurrence, development, and outcome of TB granuloma and play an important role in the whole immune process of TB infection. Macrophages are innate immune cells that express MHC class II, which gives them the ability to initiate an adaptive immune response through T cell activation. The macrophage from spinal tissue expresses CD68 and HLA-DRA [9]. Macrophages are a heterogeneous group of cells that can be divided into classical activated macrophages (M1) and alternatively activated macrophages (M2), which play a proinflammatory (M1) and an anti-inflammatory (M2) effect [10]. Studies have found that macrophage polarization is involved in the occurrence and development of TB [11, 12]. However, the role of macrophage polarization and related cytokines in spinal TB has not been clarified. Here, in this study, we focused on spinal TB and investigated the expression of different polarization types and related cytokines in macrophages to further understand the disease progression of spinal TB.

## 2. Materials and Methods

### 2.1. Subjects

This is a descriptive observational study. Thirty-six patients with spinal TB were included, including 17 males and 19 females with an average age of 56.2 years (age 4-77 years). Samples were taken from postoperative lesions, distant paraspinal cartilage tissue, and connective tissue of spinal TB patients treated in the spine surgery department of two general hospitals in Urumqi, Xinjiang, from Jan 2017 to Dec 2018, and peripheral blood was collected at the same time. Meanwhile, healthy subjects (*n* = 25) from Jan 2017 to Dec 2018 in two hospitals were enrolled as the control group, and peripheral blood was collected.

The diagnostic criteria for spinal TB are as follows: (1) patients with typical symptoms of tuberculous infection, including low fever, night sweats, weight loss, and fatigue; (2) patients with symptoms of spinal cord compression, including pain, myodynamia, muscle tension, tendon reflexes, limited activity, and spinal deformities; (3) patients with positive *M.tb* antibody; (4) patients with tuberculous granuloma; (5) patients with the typical features of spinal TB on imageology, including bone marrow edema, endplate erosion, vertebral destruction, and spinal compression; and (6) exclude patients with other immune, neoplastic diseases, and HIV infection.

All patients provided written consent to use their specimens for research purposes; none of them was identifiable. The study was approved by the ethics committee of Xinjiang Medical University.

### 2.2. Antibodies

The antibodies used in our research were used for immunohistochemistry (IHC) staining to detect the expression of the following indicators in tissues: iNOS (Abcam, UK), CD68 (Wuhan Boster, CN), Ag85B, CD163, IL-10, TNF-*ɑ*, and IFN-*γ* (Beijin Bioss, CN).

### 2.3. Tissue Preparation

The tissue samples collected in this study were bone tissue and connective tissue at the lesion site. After rinsing with saline, the tissue samples were fixed in 4% paraformaldehyde for 24-48 h. Then, the tissues were decalcified in 10% EDTA decalcification solution. Paraffin sections were obtained after ethanol gradient dehydration and embedding.

### 2.4. Ziehl-Neelsen (ZN) Staining

The tissue sections were dewaxed and stained with carbol fuchsin at 60°C for 1 h. After decolorizing with hydrochloric acid and ethanol for 30-60 secs, the tissue sections were restained with methylene blue for 1 min. After rinsing with running water, the tissue sections were then observed under an oil microscope.

### 2.5. Hematoxylin and Eosin (H&E) Staining

H*&*E staining was performed according to routine procedure: hematoxylin staining for 1 min, eosin staining for 3 min, 1% hydrochloric acid ethanol differentiation, PBS blue, ethanol gradient dehydration, neutral gum seal, and microscopic observation of tissue morphology.

### 2.6. Immunohistochemistry (IHC) Staining

The tissue sections were subjected to pepsin antigen repair after incubation with 3% hydrogen peroxide at room temperature. Then, the sections were blocked with goat serum. After that, the primary antibody (Ag85B, 1 : 300; CD68, 1 : 200; iNOS, 1 : 800; CD163, 1 : 600; IL-10, 1 : 400; TNF-*α*, 1 : 600; and IFN-*γ*, 1 : 50) was added, and samples were incubated at 4°C overnight. After rinsing with saline, the secondary antibody was added and incubated at room temperature for 1 h. The samples were finally developed with DAB chromogen. After counter staining with hematoxylin, the samples were observed under a microscope. IHC staining positive expression area was processed by ImageJ.

### 2.7. RT-PCR

EDTA anticoagulated peripheral blood (2 ml) was taken from each participant, and the red blood cells were broken by Lysate. Total RNA was extracted from the leukocyte by a TRIzol reagent (Invitrogen, US). RNA concentration (>80 ng/*μ*l) and purity (OD260/OD280 = 1.8‐2.0) were determined by a nucleic acid quantifier. cDNA was synthesized by reverse transcription according to the reagent instructions (Takara, Japan). The RT-PCR reaction system was as follows: 2 *μ*l cDNA, 1 *μ*l forward primers and reverse primers, 12.5 *μ*l TB Green Premix Ex Taq, and 8.5 *μ*l DEPC treated water. Primer sequences are shown in Table 1. GAPDH was used as the internal control. The mRNA expression levels obtained were analyzed by the comparative cycle threshold method (2^-*ΔΔ*t^). Regardless of the spinal TB group or the control group, the average value of the *Δ*CT value of the control group was used as the reference standard, that is, the spinal TB group ΔΔCT = spinal TB group CT − control group ΔCT average and the control group ΔΔCT = control group CT − control group ΔCT average. Then, use (2^-*ΔΔ*t^) to get the final data for statistical analysis.

### 2.8. ELISA

Serum samples were taken from each subject's yellow blood collection tube (containing separating glue and coagulant accelerator). Serum levels of IL-6, TNF-*α*, and IFN-*γ* were detected in accordance with ELISA kit instructions (eBioscience, Austria). The absorbance (A) was detected at a single wavelength of 450 nm. The cytokine concentration was determined according to the standard curve.

### 2.9. Statistical Analysis

All experimental data in this study were statistically analyzed by SPSS 22.0 and GraphPad Prism 8. Quantitative data were expressed as mean ± SD. The *t*-test was used to compare the differences between groups. *P* < 0.05 was considered as statistically significant.

## 3. Results

### 3.1. Clinical Data Analysis

The computed tomography (CT) and magnetic resonance imaging (MRI) results of spinal TB were observed, and the following cases were found: (1) column destruction; (2) vertebral collapse; (3) large abscess collection with a thin abscess wall, multiple vertebral involvement, end plate erosions, and intervertebral disc involvement, between the two groups; and (4) uncommon paravertebral tissue shadow and bone chelation and hardening. We found that most patients with spinal TB were over 40 years old, regardless of their occupation, gender, ethnicity, and so on. Patients were admitted to hospital due to chest and back pains or paralysis of both lower limbs. X-ray suggested that there were different degrees of vertebral destruction or bone hyperplasia at the site of lesion, as well as paravertebral shadows because of a paraspinal abscess. The C-reactive protein (CRP) level in spinal TB patients was higher than that in the control group; however, due to the nonacute phase of spinal TB, it is rare to see the increase of leukocyte (Table 2).

### 3.2. ZN Staining Results of Spinal TB Tissue

**Figure 1:**
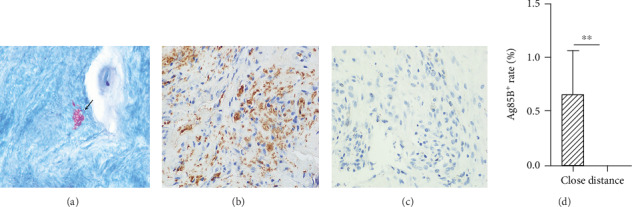
ZN staining and IHC in the TB lesion tissue. (a) ZN staining in tuberculous granuloma (×1000 oil immersion). (b) Ag85B is close to the TB lesion tissue, and the positive area is brown (×400). (c) Ag85B distance to the TB lesion tissue and negative expression (×400). (d) Quantitative comparison of Ag85B-positive area, ^∗∗^*P* < 0.01.

In ZN staining, a large number of acid-fast bacilli (AFB) were observed around and inside the tuberculous granuloma, which were about 3 *μ*m in length. The acid-fast bacilli were slender, red-stained, nonrefractive, and slightly curved, and they were specifically distributed in caseous necrosis or inside the macrophages (Figure 1(a)).

### 3.3. IHC of Ag85B

Ag85B is the most abundant protein expressed by *M.tb*. It is a myocolyc transferase in the myc pathway and catalyses—like Ag85A and Ag85C—the transfer of the fatty acid mycolate from one trehalose monomycolate to another, resulting in trehalose dimycolate and free trehalose which help build the cell wall [13]. As shown in Figure 1(b), positive staining for Ag85B was shown as brown granules distributed in the necrotic part of the lesion, Langerhans multinucleated giant cells, and epithelial-like cells. Compared with the distant tissue, the close tissue had significantly higher Ag85B expression (*P* < 0.01) (Figures 1(c) and 1(d)), which was consistent with the results of ZN staining. Since the positive range of IHC was more obvious and extensive than the ZN staining, we used both methods to increase the positive rate.

### 3.4. H&E Staining

**Figure 2:**
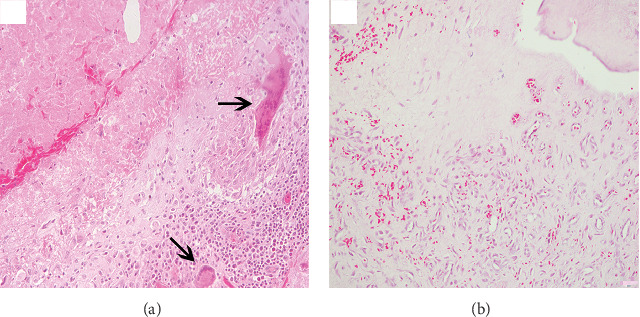
H&E staining close to the TB lesion tissue and the distance to the TB lesion tissue. (a) Close to the TB lesion tissue H&E staining, with arrows pointing to the typical Langerhans multinucleated giant cells (×200). (b) Distance to the TB lesion tissue H&E staining (×200).

In H*&*E staining, a typical tuberculous granuloma was observed in close tissue. In the center of it, we found the caseous necrosis, surrounded by radially arranged epithelioid cells, as well as a large number of macrophages and Langerhans multinucleated giant cells. Further, lymphocytes infiltrated in its outermost layer accompanied by occasional fibrous tissues. Langerhans multinucleated giant cells are typical multinucleated giant cells in tuberculous granulomas. Their morphology was mostly horseshoe-shaped, large in size, rich in cytoplasm, and pink in pulp (Figure 2(a)). The distant tissue occasionally showed inflammatory infiltration or hemorrhage (Figure 2(b)).

### 3.5. IHC Analysis of Macrophage Polarization Characteristic Molecules

**Figure 3:**
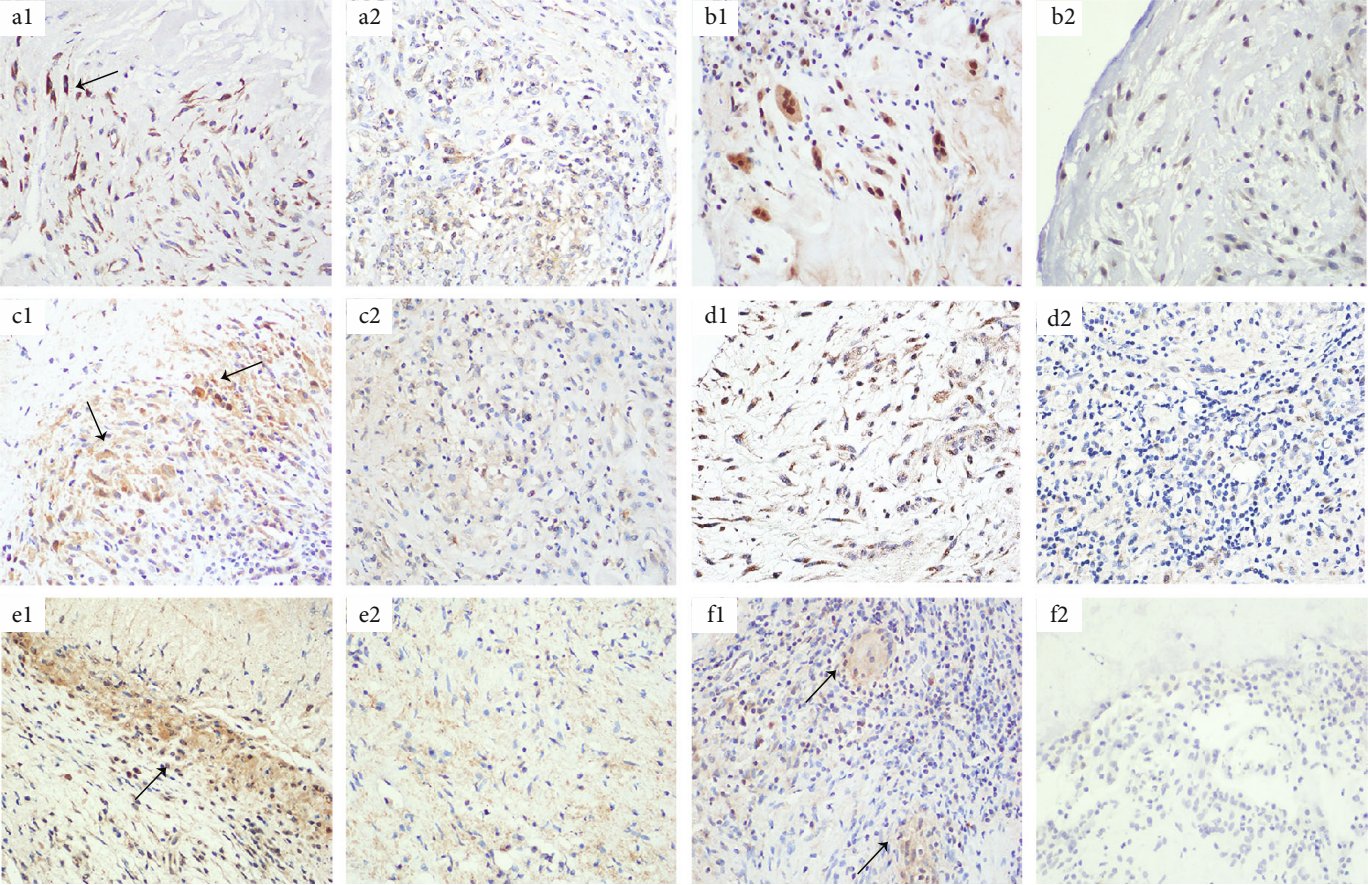
The IHC of macrophage polarization characteristic molecules and cytokines. (a1) IL-10 is close to the TB lesion tissue, and the positive area is brown (×400). (a2) IL-10 distance to the TB lesion tissue and negative expression (×400). (b1) TNF-*α* is close to the TB lesion tissue, and the positive area is dark yellow (×400). (b2). TNF-*α* distance to the TB lesion tissue and negative expression (×400). (c1) IFN-*γ* is close to the TB lesion tissue, and the positive area is dark yellow (×400). (c2) IFN-*γ* distance to the TB lesion tissue and negative expression (×400). (d1) CD68 is close to the TB lesion tissue, and the positive area is brown (×400). (d2) CD68 distance to the TB lesion tissue and the positive area is light yellow (×400). (e1) iNOS is close to the TB lesion tissue, and the positive area is brown (×400). (e2) iNOS distance to the TB lesion tissue and the positive area is light yellow (×400). (f1) CD163 is close to the TB lesion tissue, and the positive area is dark yellow (×400). (f2) CD163 distance to the TB lesion tissue and the positive area is light yellow (×400).

**Figure 4:**
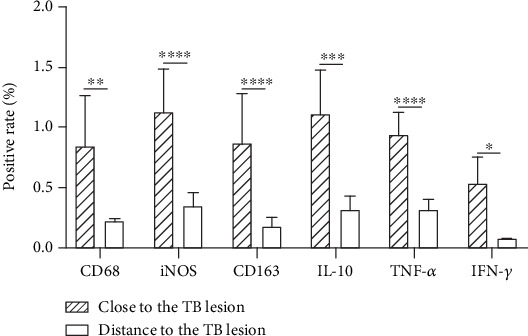
The IHC positive rates of macrophage polarization characteristic molecules and cytokines. Quantitative comparison of CD68-positive area, ^∗∗^*P* < 0.01. Quantitative comparison of iNOS-positive area, ^∗∗∗∗^*P* < 0.0001. Quantitative comparison of CD163-positive area, ^∗∗∗∗^*P* < 0.0001. Quantitative comparison of IL-10-positive area, ^∗∗∗^*P* < 0.001. Quantitative comparison of TNF-*α*-positive area, ^∗∗∗∗^*P* < 0.0001. Quantitative comparison of IFN-*γ*-positive area, ^∗^*P* < 0.05.

IHC of CD68 showed brownish granules around tuberculous caseous necrosis and positive expression on a macrophage membrane in the close tissue compared to the distant tissue (Figure 3(a1) and (a2)). Nitric oxide synthase (iNOS) is a kind of catalytic enzyme induced by injury or infection. After activation of iNOS, a large number of NO were produced to enhance the degree of oxidative stress, promote the expression of inflammatory factors, and thus eliminate bacteria [14]. iNOS, as a characteristic molecule, was positive in the cytoplasm or nucleus of M1 macrophages around the lesion (Figure 3(b1)). CD163 is expressed in most M2 macrophages and has an anti-inflammatory effect, contrary to iNOS' s proinflammatory effect. CD163 showed brown-yellow particles (Figure 3(c1)), both of which were mainly distributed in the cytoplasm of macrophages surrounding the caseous necrotic tissue and occasionally in the Langerhans multinucleated giant cells. However, iNOS and CD163 in the distant tissue were weakly positive (Figure 3(b2) and (c2)). The expression levels of macrophage-related molecules in the positive expression area (%) were significantly higher when compared with the distant tissue (Figure 4).

### 3.6. Macrophage Polarization-Related Cytokines

**Figure 5:**
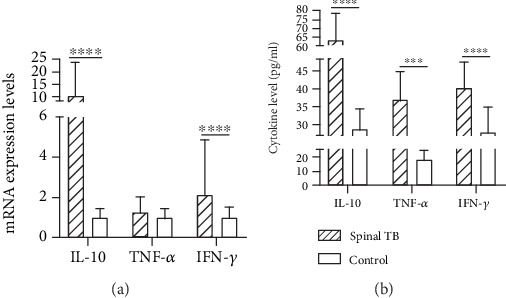
IL-10, TNF-*α*, and IFN-*γ* mRNA expression levels and cytokine levels. (a) The mRNA expression of IL-10, TNF-*α*, and IFN-*γ* in the leukocyte of spinal TB patients and control subjects. Significant differences: ^∗^*P* < 0.05 and ^∗∗∗∗^*P* < 0.0001. (b) Serum levels of IL-10, TNF-*α*, and IFN-*γ* in 36 patients with spinal TB and 25 control subjects. ^∗∗∗^*P* < 0.001 and ^∗∗∗∗^*P* < 0.0001.

We collected peripheral blood from 36 patients with spinal TB and 25 healthy controls. The expression of macrophage polarization-related cytokines was analyzed from mRNA and serum levels. TNF-*α* and IFN-*γ* are the main related factors of M1 macrophages and IL-10 is the main related factor of M2 macrophages. RT-PCR showed that in both groups, the mRNAs of IL-10, TNF-*α*, and IFN-*γ* were expressed in the leukocyte of the peripheral blood, and their levels in spinal TB patients were higher than those in the control group. Among them, the increase of IL-10 and IFN-*γ* was more obvious and statistically significant. (Figure 5(a)). The levels of serum IL-10, TNF-*α*, and IFN-*γ* in the spinal TB group were higher than those in the control group, and the differences were statistically significant (*P* < 0.01) (Figure 5(b)).

We found high levels of IL-10, TNF-*α*, and IFN-*γ* in the peripheral blood. Therefore, we collected close to the TB lesion tissue and at a distance to the TB lesion tissue from patients with spinal TB for IHC staining, to further confirm the expression of macrophage polarization-related cytokines in spinal TB. The results of IL-10 showed brownish granules on the cell membrane around the caseous necrosis (Figure 3(d1)). TNF-*α*- and IFN-*γ*-positive staining showed brown-yellow granules in the cytoplasm surrounding the necrosis (Figure 3(e1) and (f1)). They were all positive in macrophages and Langerhans multinucleated giant cells, while they were expressed as weakly positive at a distance to the TB lesion tissue (Figure 3(d2), (e2), and (f2)). The results of macrophage-associated cytokines were statistically significant compared with the distance to the TB lesion tissues (Figure 4).

## 4. Discussion

In the present study, we analyzed the type of macrophage polarization in surgical specimens from 36 patients diagnosed with spinal TB. By analyzing the clinical data of each patient, we found that patients with spinal TB were admitted to the hospital with clinical features such as thoracolumbar pain. Some patients also showed symptoms such as numbness and weakness in both lower extremities. These results indicate that the quality of life of patients with spinal TB in the early stages of the disease is reduced. At the same time, CRP and monocytes in the blood of these patients increased during the first period of hospitalization. It could be explained that in patients with spinal TB requiring surgery, the human body may have an acute response to the reproduction and destruction of *M.tb*. Therefore, it is necessary to understand the degree of disease immunity by studying whether macrophage polarization is involved in the disease process.

The *M.tb* infection is regulated by both immune and pathogenic factors [15, 16]. The research on the body immunity mainly focuses on the regulation of various cytokines on tuberculous granulomas and the function and type of T cells in the development of tuberculous granulomas. On the other hand, research on pathogens mainly focuses on the mechanism of induction and intervention of tuberculous granuloma formation, such as virulence factors. The mannose receptor on macrophages can mediate their binding to the arabinomannan on *M.tb*, and thus, *M.tb* is phagocytized [17]. Previous studies [18] have shown that alveolar macrophages play a role in clearing *M.tb* in lung TB, but the role of macrophages in spinal TB has rarely been reported.

Macrophages serve dual roles in the pathogenesis of TB in that macrophages are both the predominant host cell and the first line of defense against *M.tb* infection. We used IHC staining to detect the expression of macrophage-specific molecules in the lesion tissue and found that CD68 was positively expressed in the surrounding cells of the caseous necrosis; CD68 is a characteristic marker of macrophage [19], suggesting a large number of macrophage infiltration. Around the caseous necrosis, *M.tb* secreted a large amount of toxic substances. Macrophages, which are the main effector cells for killing bacteria, gathered around the lesions and played a role in killing tubercle bacilli.

At present, the concept of M1/M2 macrophage polarization, albeit oversimplified and based primarily on in vitro data, still remains one of the best means by which macrophage activation can be described. M1 macrophages function at the crux of host defense by eliciting essential proinflammatory responses and bridging innate and adaptive immunities. M1 macrophages mainly release proinflammatory factors such as tumor necrosis factor-*α* (TNF-*α*) and interleukin-6 (IL-6) and kill *M.tb* infection at an early stage by phagocytosis, presenting as an antigen and initiating adaptive immune response. In our study, iNOS, as a M1 macrophage-specific molecule, has a higher expression in tuberculosis lesion tissue. It may be that when *M.tb* infects the spine, M1 macrophages play a part in the proinflammatory effect and start the host defense mechanism.

TNF-*α* [20] is a cytokine produced by M1 macrophages and has promoting effects on cytokines in leukocytes, vascular endothelial cells, and connective tissues. At the same time, TNF-*α* is also a bone resorption inducer, which can promote the differentiation and absorption of osteoclasts by inducing osteoblast expression of NF-*κ*B receptor activator ligand and M-CSF, thereby inhibiting spinal TB lesions. However, elevated expression of TNF-*α* may damage the cancellous bone of the vertebral body in TB patients, resulting in degeneration and necrosis of the intervertebral disc, which is one of the causes of the disappearance of the intervertebral space. IFN-*γ* is an important factor in anti-*M.tb* infection. Its high expression in the serum can effectively inhibit the growth and spread of intracellular *M.tb*.

In contrast, M2 macrophages are crucial to immune regulation by promoting the resolution of inflammation and preventing an exacerbated, chronic inflammatory state, as well as the maintenance of tissue homeostasis by inducing tissue healing and remodeling [21]. M2 macrophages inhibit inflammatory responses by secreting interleukin-10 (IL-10) and play a role in immune tolerance and repair of damaged tissues. CD163 has essentially a homeostatic activity. CD163 has been recently associated with M2 polarization of macrophages. In particular, CD163 was considered as a marker of the M2a and the M2c polarization status [22]. In the present work, a significant increase in CD163-positive macrophages was observed in spinal TB lesion tissues. We found that the expression level of IL-10 was significantly increased in the spinal TB group, which was consistent with the increase of M2 macrophages. IL-10 exerts resistance and clearance to *M.tb* in the host by regulating immune balance, releasing immune mediators, and presenting antigens.

iNOS and CD163 are involved in two opposite activities, proinflammatory (M1) versus anti-inflammatory (M2) function. The interplay between the iNOS and CD163 pathways is complex. In Figure 5, here we report that iNOS (M1 marker) and CD163 (M2 marker) were highly expressed in the lesion tissues, indicating that there are two different types of macrophages in the tuberculous granulomas of spinal TB patients, exerting both proinflammatory and anti-inflammatory effects [23]. M1 macrophages produce inflammatory factors and activate adaptive immunity. M2 macrophages have an inhibitory effect on immune responses, inhibiting antigen presentation and T cell proliferation, exerting anti-inflammatory responses, attenuating cellular immunity against TB infection, and thereby promoting chronic TB infection [24].

## 5. Conclusion

The mechanism by which tuberculous granuloma causes bone destruction is complex. In this study, we found that macrophage polarization was closely related to infection of spinal TB. Our research shows that in patients with spinal TB requiring surgery, there are mainly two types of macrophage polarization (M1 and M2), which play a proinflammatory and an anti-inflammatory role, respectively. Both are coordinated and antagonized. By studying the type of macrophage polarization, we can observe the degree of disease in spinal TB that requires surgery. In order to further understand how the disease develops, in future research, we hope that we can study the mechanism and learn more about the spinal TB.

## Figures and Tables

**Table 1:** Primer sequence.

Name	Primer sequence
IL-10	Forward ATCCAAGACAACACTACTAA
	Reverse TAAATATCCTCAAAGTTCC
TNF-*ɑ*	Forward TGCTCCTCACCCACACCAT
	Reverse GGAGGTTGACCTTGGTCTGGTA
IFN-*γ*	Forward CTAATTATTCGGTAACTGACTTGA
	Reverse ACAGTTCAGCCATCACTTGGA
GAPDH	Forward CATCCACTGGTGCTGCCAAGGCTGT
	Reverse ACA ACCTGGTCCTCAGTGTAGCCCA

Note: IL-6: interleukin-6; TNF-*α*: tumor necrosis factor-*α*; IFN-*γ*: interferon-*γ*; GAPDH: glyceraldehyde-3-phosphate dehydrogenase.

**Table 2:** Clinical data of spinal TB group and control group.

	Experimental *n* = 36	Control *n* = 25
Age	56.20 ± 5.80	44.20.±11.50
Gender		
Male	17 (47%)	12 (48%)
Female	19 (53%)	13 (52%)
Ethnicity		
Han	8 (22%)	7 (28%)
Minority	28 (78%)	18 (72%)
Occupation		
Labor-type	10 (28%)	6 (24%)
Nonlabor type	17 (47%)	12 (48%)
Freelance	9 (25%)	7 (28%)
Risk factor		
History of TB	4 (11%)	0
HIV	0	0
Main symptoms		
Numbness of both lower limbs	10 (28%)	0
Neuropathic pain	35 (97%)	0
Systemic symptoms	11 (31%)	0
X-ray		
Column destruction	16 (44%)	0
Paravertebral tissue shadow	14 (39%)	0
Bone chelation and hardening	1 (2%)	0
Osteosis	1 (2%)	0
Biochemical analysis		
Increased T-SPORT	16 (44.4%)	0
Increased CRP	22 (61%)	0
Increased leukocyte	2 (6%)	1 (4%)
Increased M	25 (69.4%)	1 (4%)

Note: TB: tuberculosis; HIV: Human Immunodeficiency Virus; CRP: C-reaction protein; T-SPOT.TB: T cell spot test tuberculosis; M (Monocytes).

## Data Availability

The data used to support the findings of this study are included within the article.

## References

[1] López-Ramos J. E., Macías-Segura N., Cuevas-Cordoba B. (2018). Improvement in the diagnosis of tuberculosis combining *mycobacterium tuberculosis* immunodominant peptides and serum host biomarkers. *Archives of Medical Research*.

[2] Schutz C., Barr D., Andrade B. B. (2019). Clinical, microbiologic, and immunologic determinants of mortality in hospitalized patients with HIV-associated tuberculosis: a prospective cohort study. *PLoS Medicine*.

[3] Chhawra S., Jain R., Aggarawal R., Pandey A. (2019). A rare case of radius head epiphyseal aneurysmal bone cyst with predisposing factor as trauma tuberculosis of elbow apart from genetic. *Journal of Orthopaedic Case Reports*.

[4] Ma J., Lv Z., Wang J., Lu J. (2019). Relationship between IL-10 gene polymorphism and spinal tuberculosis. *Medical Science Monitor*.

[5] Kasempimolporn S., Thaveekarn W., Promrungreang K., Khow O., Boonchang S., Sitprija V. (2017). Improved serodiagnostic sensitivity of strip test for latent tuberculosis. *Journal of Clinical and Diagnostic Research*.

[6] Orme I. M. (2016). Vaccines to prevent tuberculosis infection rather than disease: physiological and immunological aspects. *Tuberculosis (Edinburgh, Scotland)*.

[7] Ginsberg A. M., Ruhwald M., Mearns H., McShane H. (2016). TB vaccines in clinical development. *Tuberculosis*.

[8] Marino S., Hult C., Wolberg P., Linderman J. J., Kirschner D. E. (2018). The role of dimensionality in understanding granuloma formation. *Computation (Basel)*.

[9] Wood M. J., Leckenby A., Reynolds G. (2019). Macrophage proliferation distinguishes 2 subgroups of knee osteoarthritis patients. *JCI Insight*.

[10] Tang Y., Zhao L., Lei N., Chen P., Zhang Y. (2019). Crohn's disease patients with depression exhibit alterations in monocyte/macrophage phenotype and increased proinflammatory cytokine production. *Digestive Diseases*.

[11] Andersson A. M., Larsson M., Stendahl O., Blomgran R. (2019). Efferocytosis of apoptotic neutrophils enhances control of *mycobacterium tuberculosis* in HIV-coinfected macrophages in a myeloperoxidase-dependent manner. *Journal of Innate Immunity*.

[12] Gao X., Wu C., Wang X. (2019). The DosR antigen Rv1737c from *Mycobacterium tuberculosis* confers inflammation regulation in tuberculosis infection. *Scandinavian Journal of Immunology*.

[13] Che N., Qu Y., Zhang C., Zhang L., Zhang H. (2016). Double staining of bacilli and antigen Ag85B improves the accuracy of the pathological diagnosis of pulmonary tuberculosis. *Journal of Clinical Pathology*.

[14] Nadella V., Ranjan R., Senthilkumaran B. (2019). Podophyllotoxin and rutin modulate M1 (iNOS+) macrophages and mitigate lethal radiation (LR) induced inflammatory responses in mice. *Frontiers in Immunology*.

[15] Kim W. S., Jung I. D., Kim J. S. (2018). *Mycobacterium tuberculosis* GrpE, a heat-shock stress responsive chaperone, promotes Th1-biased T cell immune response via TLR4-mediated activation of dendritic cells. *Frontiers in Cellular and Infection Microbiology*.

[16] Khan A., Singh V. K., Hunter R. L., Jagannath C. (2019). Macrophage heterogeneity and plasticity in tuberculosis. *Journal of Leukocyte Biology*.

[17] Mehra S., Alvarez X., Didier P. J. (2013). Granuloma correlates of protection against tuberculosis and mechanisms of immune modulation by *Mycobacterium tuberculosis*. *The Journal of Infectious Diseases*.

[18] Rothchild A. C., Olson G. S., Nemeth J. (2019). Alveolar macrophages generate a noncanonical NRF2-driven transcriptional responseto *Mycobacterium tuberculosis* in vivo. *Science Immunology*.

[19] Kono Y., Saito H., Miyauchi W. (2020). Increased PD-1-positive macrophages in the tissue of gastric cancer are closely associated with poor prognosis in gastric cancer patients. *BMC Cancer*.

[20] Ruiz A., Guzmán-Beltrán S., Carreto-Binaghi L. E., Gonzalez Y., Juárez E. (2019). DNA from virulent *M. tuberculosis* induces TNF-*α* production and autophagy in M1 polarized macrophages. *Microbial Pathogenesis*.

[21] Lisi L., Ciotti G. M. P., Braun D. (2017). Expression of iNOS, CD163 and ARG-1 taken as M1 and M2 markers of microglial polarization in human glioblastoma and the surrounding normal parenchyma. *Neuroscience Letters*.

[22] Isohisa T., Asai J., Kanemaru M. (2020). CD163-positive macrophage infiltration predicts systemic involvement in sarcoidosis. *Journal of Cutaneous Pathology*.

[23] Chen X., Yang B., Tian J. (2018). Dental follicle stem cells ameliorate lipopolysaccharide-induced inflammation by secreting TGF-*β*3 and TSP-1 to elicit macrophage M2 polarization. *Cellular Physiology and Biochemistry*.

[24] Refai A., Gritli S., Barbouche M. R., Essafi M. (2018). Mycobacterium tuberculosis virulent factor ESAT-6 drives macrophage differentiation toward the pro-inflammatory M1 phenotype and subsequently switches it to the anti-inflammatory M2 phenotype. *Frontiers in Cellular and Infection Microbiology*.

